# Current Status and Evaluation of Randomized Clinical Trials of Traditional Chinese Medicine in the Treatment of Cardiovascular Diseases

**DOI:** 10.1155/2022/6181862

**Published:** 2022-01-12

**Authors:** Xi Guo, Xiaojun Chen, Jinlan Chen, Zhiping Tan, Yifeng Yang, Hong Zhang

**Affiliations:** ^1^Department of Cardiovascular Surgery, The Second Xiangya Hospital, Central South University, No. 139 Middle Renmin Road, Changsha, Hunan 410011, China; ^2^Department of Nephrology, The Second Xiangya Hospital, Central South University, No. 139 Middle Renmin Road, Changsha, Hunan 410011, China

## Abstract

Traditional Chinese medicine has long been applied to various diseases in China for a few thousand years. In recent years, its market has gradually developed from Asian countries to Western countries. At present, due to the lack of evidence-based medicine research, the effect of traditional Chinese medicine on the prevention and treatment of cardiovascular disease remains unclear. In evaluating the efficacy and safety of drugs, randomized controlled clinical trials (RCTs) are recognized as the gold standard for testing the effectiveness and safety of treatments and could offer the best evidence for the formulation of clinical treatment guidelines. Although traditional Chinese medicine has long been used to treat cardiovascular diseases, the research on the application of RCT to test the combination of traditional Chinese and Western medicine therapy or single traditional Chinese medicine therapy started late, and the number is comparably small. In order to summarize and objectively evaluate the research results of integrated traditional Chinese and Western medicine in intervention of cardiovascular diseases, we reviewed the literature of RCTs in this field by searching some Chinese and English databases and put forward some suggestions for the future development and research of traditional Chinese medicine.

## 1. Background

Cardiovascular disease (CVD) has become the leading cause of death worldwide in recent decades, accounting for 17.3 million deaths per year globally, the number of which may rise to 23.6 million by 2030 [1]. In the practice of diagnosis and treatment of CVDs in China, traditional Chinese medicine (TCM) has had wide applications for many years due to relatively insufficient medical treatment with Western medicine. Patients receiving TCM treatment tend to use Western medicines recommended by the corresponding guidelines less frequently [2]. A domestic survey of patients with Western medicine, traditional Chinese medicine, and integrated traditional Chinese and Western medicine (integrated traditional Chinese and Western medicine) showed that among the 2,712 patients who received cardiovascular disease treatment, only 3.1% received traditional Chinese medicine treatment, and 30.0% integrated medicine treatment. And 66.9% only received Western medicine [3]. Contrarily, due to the lack of research on its mechanism and effectiveness, Chinese medicine is implemented as a complementary or alternative therapy. Moreover, TCM is often criticized or rejected because that few high-quality evidence-based testimonies can meet evaluation criteria. To this end, a large number of basic and clinical studies have been performed to validate the efficacy of TCM. In this review, we summarize the current status and progress of randomized controlled trials (RCTs) on the efficacy and safety of TCM in the treatment of some CVD risk factors (hypertension and dyslipidemia) or 2 major types of CVDs: atherosclerotic cardiovascular disease (ASCVD), which is also defined as coronary artery disease [4], and chronic heart failure.

## 2. Literature Sources and Search Strategies

This study retrieved 7 Chinese and English electronic databases, including China HowNet (CNKI), Wanfang Data Knowledge Service Platform (Wanfang), VIP Journal Full-Text Database (VIP), Chinese Biomedical Literature Database (SinoMed), PubMed, and Cochrane Library. In addition, we also searched China Clinical Trial Registration Center (http://www.chictr.org.cn) and the U.S. Clinical Trials Center (https://clinicaltrials.gov). Since many clinical studies on traditional Chinese medicine are published in Chinese, we have collected both Chinese and English literature. The search algorithm for database was as follows: (“traditional Chinese medicine” OR “TCM”) AND (“cardiovascular disease” OR “hypertension” OR “bloodpressure” OR “dyslipidemia” OR “hyperlipidemia” OR “lipid” OR “atherosclerotic cardiovascular disease” OR “ischemic heart disease” OR “coronary heart disease” OR “coronary artery disease” OR “myocardial infarction” OR “heart failure”) AND (“randomized” OR “randomized controlled trial”) AND “blind”. We adjusted the specific searching strategies according to different features of every database without other restrictions. The search scope of database and clinical trials center is 8/1/2011 to 8/1/2021.

We included clinical research literature according to the following criteria: (1) Patients are diagnosed with CVDs we mentioned above. (2) The sample size of study group should be more than 50 cases. (3) Intervention measures include all types of traditional Chinese medicine, such as traditional Chinese medicine, acupuncture, massage, Guasha, cupping, qigong, moxibustion, and other treatments based on theories of TCM. (4) The type of literature research is randomized controlled trial. Reports complied following criteria were excluded: (1) nonrandomized controlled or nonblinded trials. (2) The diagnosis of the recruited patients was not clear. (3) Reports with Jadad score <3. (4) Any report without objective laboratory examinations. (5) Reports involving the comparison between a variety of TCM intervention measures were excluded. In addition, reports that describe the experimental methods and procedures that are too simplistic were also excluded. (6) For similar trials, we chose reports with larger samples and more adequate results.

## 3. Literature Search Results

**Figure 1:**
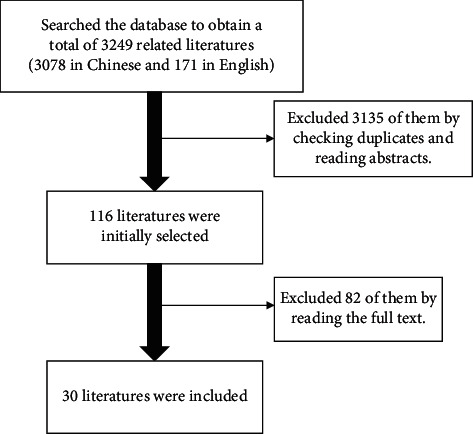
Literature screening process.

Through the above search strategies, we initially retrieved 3249 relevant Chinese and English literatures, of which 3078 were Chinese literatures and 171 were English literatures. By duplicate checking and reading abstracts, 114 articles were screened out. Read the full text for rescreening, and finally 30 literatures were included, including 16 in English and 14 in Chinese. The literature screening process is shown in Figure 1.

The database was searched to obtain a total of 3249 related literatures (3078 in Chinese and 171 in English). The database was researched to obtain a total of 3000 related documents.

### 3.1. TCM for Risk Factors of CVD

#### 3.1.1. Hypertension

So far, several TCM interventions for hypertension have been reported, including Tiankuijiangya tablet, Gastrodia-Uncaria granules, Jiangyabao tablet, Bushen Qinggan formula, Qiqilian capsule, Jingui Shenqi pill, and TCM acupuncture. The basic characteristics of the included studies are shown in Table 1. Among these treatments, it is noted that Gastrodia-Uncaria granules (GUG) have a significant effect on patients with masked hypertension [5]. The daytime systolic BP (SBP)-diastolic BP (DBP) of the Gastrodia-Uncaria granules and the placebo group were reduced by 5.44/3.39 and 2.91/1.60 mmHg, respectively. The daytime BP and 24-hour BP alleviated significantly (*P* < 0.05), but the clinic BP and night BP were not improved significantly. The per-protocol analysis in 229 patients showed similar results. Its safety has been confirmed, since only 1 adverse event (drowsiness during the day) was reported and no serious adverse event was reported. The efficacy and safety of Tiankuijiangya tablet was examined in a multicenter clinical trial [6]. The significant efficacy was 63.23% in the experimental group and 31.65% in the control group. The significant efficiency and effective rate of the TCM syndrome were 48.39% and 20.25%, respectively, in the experimental group, while those in the control group were 20.25% and 36.71%, respectively. There were no serious adverse reactions were found in both groups. Bushen Qinggan formula was proved to influence the control of blood pressure variability (BPV) and endothelial markers on the basis of standard treatments (amlodipine); though it did not show a significant antihypertensive effect [7]. A total of 150 patients with hypertension were randomly divided into three groups: placebo, Bushen Qinggan decoction, and Bushen Qinggan granule, and all the three groups were treated with standard drug (amlodipine). Blood pressure deviation (BPD) and blood pressure variability (BPV) both decreased significantly compared with the control group. In another study, after 4-week treatment of Qiqilian capsule combined with extended release nifedipine tablets, a significant difference was observed between treatment group and placebo group [8]. In this study, 118 patients with hypertension were randomly divided into two groups: treatment group (*n* = 54) and control group (*n* = 64). Both groups were treated with nifedipine sustained-release tablets; the treatment group was treated with Qilian capsule, and the control group was treated with placebo. The results showed that the antihypertensive effect and effective rate of the treatment group were better than those of the control group (both *P* < 0.05). Jingui Shenqi pill has a positive effect on hypertension and lipid metabolism [9]. In this study, 103 elderly patients with hypertension were randomly divided into study group (*n* = 52) and control group (*n* = 51). The study group was treated with Jingui Shenqi pills combined with nifedipine controlled-release tablets, and the control group was treated with nifedipine controlled-release tablets. In contrast to the control group in the 4th week after treatment, BP in the study group was significantly reduced (*P* < 0.01). After treatment, the TC, TG, LDL-C, and HDL-C in the study group were improved, which were significantly different from those in the control group before treatment (*P* < 0.05). There were no serious adverse reactions in both groups. Jiangyabao tablet combined with Western medicine (nifedipine sustained-release tablets and hydrochlorothiazide) is effective in improving nocturnal blood pressure in young and middle-aged patients with grade 2 hypertension [10]. In this study, 199 patients aged 30–60 years old were randomly assigned into treatment group and control group, of which 99 were in the treatment group and 100 were in the control group. Both groups were treated with nifedipine sustained-release tablets and hydrochlorothiazide tablets. On this basis, the treatment group was treated with combined Jiangyabao tablet, while the control group was treated with placebo, both 0.31 g/tablets. The course of treatment was 8 weeks, and the follow-up period was 24 weeks. The change of 24-hour ambulatory BP and occasional BP were measured and evaluated between the two groups; adverse reactions were also recorded. Compared with that before treatment, the day and night occasional BP and 24-hour ambulatory BP in both groups were significantly refined after treatment. The average decrease of nocturnal systolic blood pressure and diastolic blood pressure in the treatment group was greater than that in the control group, and the difference was statistically significant (*P* < 0.05). The improvement of occasional blood pressure and ambulatory blood pressure in the treatment group was not statistically significant in contrast to the control group (*P* > 0.05).

Acupuncture was also proved to be effective for mild hypertension [11]. A trial included 428 patients with SBP between 140 mm Hg and 159 mm Hg and/or DBP (DBP) between 90 and 99 mmHg. The patients were randomly divided into 4 groups (107 cases in each group). They received the affected acupoints acupuncture, unrelated acupoints acupuncture, and sham acupuncture or were moved to the waiting-list control group. The ambulatory BP of all patients was monitored at the 6th, 9th, and 12th weeks. At the 6th week, the SBP in the acupuncture group was lower than that in the sham group and waiting-list control group, but the difference was not significant (both *P* > 0.05). However, at the 9th week, the acupuncture group was better than the sham group (3.3 mmHg, *P* < 0.05) and the waiting-list control (4.8 mmHg, *P* < 0.001). The results show that acupuncture has a slight antihypertensive effect on patients with mild hypertension.

The above evidence indicates that some Chinese medicines have clear curative effects on hypertension and could be used as alternative and adjuvant treatments. However, the long-term effects of these interventions on control of blood pressure and incidence of severe cardiovascular events remain unclear.

#### 3.1.2. Dyslipidemia

A total of 6 trials on treatment for dyslipidemia meet our inclusion criteria. The sample size ranges from 100 to 358, and the average follow-up time is 1 to 12 months. The basic characteristics of the included study are shown in Table 2. Most of them were compared with placebo. Based on the treatment of atorvastatin calcium tablets, the addition of Jiangzhi Tongluo soft capsules can safely reduce the serum total cholesterol (TG) level in patients with hyperlipidemia [12]. TG decreased by 26.69% and 33.29% in the 4th and 8th weeks in combined treatment group, and the decrease rates in Atorvastatin group were 25.7% and 22.98%, respectively. At the 8th week, there was a significant difference in the delta change of TG between combined treatment and control group. Though the levels of serum low-density lipoprotein cholesterol (LDL-C) and total cholesterol (TC) in the two groups were significantly reduced, no statistically significant difference in the above indicators has taken place between the two groups. Compared to placebo, Danshen (*Salvia miltiorrhiza*) and Gegen (Radix puerariae) (D&G) was proven to reduce LDL and TC in postmenopausal women [13]. After 12-month follow-up, the baseline characteristics of D&G group and placebo group are comparable. The BP and general biochemical parameters of the two groups did not change significantly. But the serum LDL-C (−6.92%) and TC (−5.85%) of the D&G group significantly reduced compared with the placebo group (−3.21% and −3.42%). A total of 12 adverse events were reported in this trial (6 in the placebo group and 6 in the D&G group), but none of them were directly related to the research herbal preparation. Xuezhitong (XZT) capsules can also significantly reduce the TG level with good safety [14]. In this research, 358 hypertriglyceridemia subjects were enrolled and randomly assigned to receive XZT (2700 mg per day), Xuezhikang (XZK) (1200 mg per day), or placebo. During the 12-week follow-up, both groups were observed to have a decrease in TG levels compared to baseline, but the decrease in the XZT and XZK groups was significantly greater than that in the placebo group (30.77%, 24.02%, and 11.59%, *P* < 0.0167); 70.54% of subjects in the XZT group and 62.30% of the subjects in the XZK group showed at least a 20% reduction in TG levels, compared to only 41.67% of subjects in the placebo group. Compared with placebo, XZT capsule treatment was effective in lipid control (17.97% vs. 5.00%) and increased HDL-C levels by ≥ 4 mg/dl. XZT is also safe and well tolerated, and no subjects developed myopathy or significantly increased liver transaminase or creatine kinase. An improved method of acupuncture, in which acupoint adopts the tapping method with plum blossom needle, was also been proven to have a possible lipid-lowering effect on obese patients with hyperlipidemia [15]. The total effective rate of the combined treatment group and the acupuncture group were 96.2% (50/52) and 84.6% (44/52), respectively, and the difference was not statistically significant. After treatment, parameters of obesity, serum lipid, lipid island axis, and autonomic nerve function became more refined in contrast to before treatment (*P* < 0.01, *P* < 0.05). Another trial proved that *Ganoderma lucidum* and sea cucumber extract can help to reduce serum TC and triglycerides in people with hyperlipidemia [16]. Patients with hyperlipidemia were selected and randomly distributed to treatment group (60 cases) and control group (60 cases). The treatment group received oral *Ganoderma lucidum* and sea cucumber extract, and the control group received a placebo for 45 days. The levels of TC and TG in the treatment group decreased significantly, and the decrease rates were 11.76 ± 6.12% and 16.63 ± 7.62%, respectively. According to Farahmand et al.'s research, the combination of cupping and dietary advice did not show a significant difference compared to dietary advice alone in patients with metabolic syndrome [17]. 136 patients between the ages of 18 and 65 participated in the trial. The experimental group was treated with wet cupping combined with diet guidance. The control group only accepted dietary advice. Anthropometric and biochemical parameters were evaluated at baseline and 6 and 12 weeks after treatment. There was no significant difference in blood lipids and anthropometric characteristics between the two groups (*P* > 0.05).

Based on the above evidence, some Chinese medicines have a certain effect on lowering serum lipids, such as Jiangzhi Tongluo soft capsule, Danshen (*Salvia miltiorrhiza*) and Gegen (Radix puerariae), Xuezhitong capsule, and *Ganoderma lucidum* and sea cucumber extract. These TCM can be used as a supplementary treatment for Western medicine in patients with hyperlipidemia. Acupuncture may have a certain effect, but the evidence is inclusive and needs further confirmation. After all, whether these beneficial effects can reduce cardiovascular events still requires further high-quality, large-scale randomized controlled trials.

### 3.2. TCM for ASCVD

ASCVD includes angina pectoris, myocardial infarction, ischemic stroke, transient ischemic attack, and peripheral artery disease. We included studies that met the above diagnostic criteria [18]. The basic characteristics of the included studies are shown in Table 3. A total of 11 studies met our standards. The number of subjects included ranges from 100 to 2674. The follow-up period ranges from 4 weeks to 2 years. Study has proved that Xinling Wan pill has significant effect on stable angina pectoris [19], in which a total of 232 subjects were included. The treatment group took Xinling Wan pills for 4 weeks, while the control group was given placebo. Effectiveness evaluation shows that Xinling pill can significantly prolong the total treadmill time of patients with stable angina. FAS analysis demonstrated the difference in total exercise time before and after treatment in the treatment group was (72.11 ± 139.32)s, and the control group was (31.25 ± 108.32)s. Xinling pill can significantly improve the total effective rate of angina pectoris symptom score; FAS analysis showed that the total effective rate of the treatment group was 78.95%, and that of the control group was 42.61%. According to FAS analysis, reduced amount of nitroglycerin was 2.45 ± 2.41 tablets in the treatment group, in contrast to 0.50 ± 2.24 tablets in the control group. It can also reduce the frequency of weekly angina pectoris, the duration of angina pectoris, etc. There were no serious adverse events in this study. The incidence of adverse events in the treatment group and the control group was 3.48% and 8.55%, respectively, with no statistical significance. Shexiang Baoxin pill (MUSKARDIA), as an additional medicine on the basis of standard treatment for patients with stable CAD, can safely reduce the frequency of angina in patients with stable CAD. In addition, MUSKARDIA has a potential effect of decreasing incidence of MACE (major adverse cardiovascular event) in patients with stable CAD [20]. A total of 2674 participants with stable CAD from 97 hospitals in China were divided into a MUSKARDIA or placebo group and observed for 24 months. The main outcome is the occurrence of MACE, defined as a composite event of cardiovascular death, nonfatal myocardial infarction (MI), or nonfatal stroke. Secondary outcomes included all-cause mortality, nonfatal myocardial infarction, nonfatal stroke, hospitalization for unstable angina or heart failure, peripheral revascularization, angina stability, and angina frequency. A total of 99.7% of patients received aspirin treatment, and 93.0% of patients received statin treatment. After 2 years of treatment, the incidence of MACE in the MUSKARDIA group was reduced by 26.9% (1.9% vs. 2.6%; odds ratio = 0.80; 95% CI: 0.45–1.07; *P* = 0.286). At the 18th month, the incidence of angina in the MUSKARDIA group was decreased with statistical significance (*P* = 0.0362). The other outcomes were similar in the two groups. The incidence of adverse events between the two groups was also comparable (17.7% vs. 17.4%, *P* = 0.8785). For stable CAD patients undergoing percutaneous coronary intervention (PCI) within 3–12 months, the combined use of Xinyue capsule with standard treatment can reduce the incidence of primary composite endpoints (cardiogenic death, nonfatal myocardial infarction, and emergency revascularization) [21]. In this trial, 1054 patients with stable CAD who had received PCI in the past 3–12 months were randomly assigned to take Xinyue capsules or placebo for 24 weeks based on standard treatment. The main endpoint is a composite endpoint, including cardiac mortality, nonfatal myocardial infarction, and emergency revascularization (PCI or CABG). Secondary composite endpoints included stroke, readmission due to acute coronary syndrome (ACS), pulmonary embolism, peripheral vascular events, and all-cause mortality. The 36-Item Short-Form Survey (SF-36) was used to assess the quality of life. The incidence of main endpoint event was 3.02% (16 patients) in Xinyue group and 6.49% (34 patients) in the placebo group (HR 0.455, 95% CI 0.25 to 0.825; *P* = 0.009), respectively. The incidence of secondary endpoint events was 5.47% in Xinyue group and 10.31% in the placebo group (HR 0.515, 95% CI 0.328 to 0.809; *P* = 0.004). Xinyue group's SF-36 score at the 12th month was significantly higher in contrast to the placebo group's. In another trial, Suxiao Jiuxin pill (SJP) was proven to reduce the MACE of ACS patients with early PCI and can improve heart function and quality of life. At the same time, it may be safe to use [22]. 200 ACS patients with early PCI were randomly assigned to SJP group (*n* = 100) and placebo group (*n* = 100). The SJP group received conventional treatment and SJP treatment. The placebo group received conventional treatment and the same amount of SJP placebo. The course of treatment was 6 months, and the follow-up was 12 months. Compared with placebo, the incidence of MACE in the SJP group was relatively low (*P* < 0.05, OR 1.916, and 95% CI 0.999–3.674). During the 360-day follow-up, the left ventricular ejection fraction (LVEF) of the SJP group was significantly higher compared to the placebo group (*P* < 0.01). SJP scored significantly higher on the SAQ subscales of physical limitation, angina frequency, and treatment satisfaction (*P* < 0.05). In contrast to placebo, the serum concentrations of Fib and cysC in SJP were significantly reduced (*P* < 0.05). Experiments confirmed that Guanxinning tablet (GXN) may have a certain positive effect on stable angina pectoris. 160 patients with stable angina pectoris were randomly assigned to receive 4 tablets (0.38 g/tablet) of GXN (80 cases) or placebo (80 cases, Guanxinning simulated tablets, the main ingredient is lactose), three times a day for 12 weeks. After the treatment, the two groups of patients were subjected to exercise stress test (treadmill program), TCM syndrome score, electrocardiogram (ECG), and nitroglycerin withdrawal rate for comparison. Compared with the control group, the length of exercise in the GXN group prolonged by 29.28 ± 17.67 s (*P* > 0.05); in addition, in the subgroup analysis, the change in the length of exercise in the GXN group increased by 63.10 ± 96.96s (*P* < 0.05). Angina pectoris, CM syndrome, ECG effective rate, and nitroglycerin withdrawal rates in the GXN group were 81.33%, 90.67%, 45.76%, and 70.73%, respectively, which were significantly higher than those in the control group (40.58%, 75.36%, 26.92%, and 28.21%, *P* < 0.05). Shuangshen Tongguan capsule (STC) can alleviate the symptoms of angina pectoris in patients with AMI after PCI and improve heart function. It has a certain effect on improving myocardial microcirculation perfusion and the quality of life [23]. In this RCT, the treatment group took STC, and the control group took STC placebo; both groups were based on standard treatment. After 6 months of treatment, the scores of TCM syndromes, heart function at rest and under stress, microcirculation perfusion rate, and Seattle Angina Pectoris Scale scores were recorded. The score of TCM syndromes decreased significantly, while the efficacy of TCM syndromes, the percentage of LVEF and normal myocardium under load, the microcirculation perfusion *k*-value before myocardial infarction increased, the total score of Seattle angina pectoris, and degree of satisfaction with treatment were significantly improved. Another study showed that the combination of Shenzhu Guanxin Recipe (SGR) and standard Western medicine treatment (SWMT) in the treatment of patients with angina after PCI may be more effective and safer than using SWMT alone [24]. The angina pectoris score (APS), CM symptom score, Seattle Angina Pectoris Questionnaire (SAQ) score, and other outcome indicators were evaluated at 1, 2, 3, and 12 months, and emergency treatments such as mortality and restenosis were observed. A more significant intratreatment effect size (*d* = 1.74) was observed in the treatment group. Compared with the control group, the APS from the pretreatment to the 12-month follow-up assessment was reduced by 76.7% (*d* = 0.83, 53.8% symptoms); the effect size between treatment (BT) is *d* = 0.66. The CM symptom evaluation of the subtreatment group decreased by 18.3% (*d* = 0.46), and the control group decreased by 16.1% (*d* = 0.31); in terms of SAQ score, the treatment group had a larger BT effect on disease awareness (*d* = 0.63), physical activity limitation (*d* = 0.62), the onset of angina (*d* = 0.55), treatment satisfaction (*d* = 0.31), and angina homeostasis (*d* = 0.30). The control group and the treatment group recorded 2 cardiovascular-related deaths and 1 accidental death, respectively. No significant differences in cardiovascular events (including deaths, hospitalization, or frequency of emergency department visits) were found between the two groups. In an experiment including 1500 patients with stable coronary artery disease, Qing-Xin-Jie-Yu Granule (QXJYG) was proven to have a potential effect on stable angina pectoris [25]. A total of 1500 recorded SCAD patients were randomly assigned to the QXJYG or placebo group at a 1 : 1 ratio for 6 months and then followed up for another 6 months. The main outcome is the composite outcome of cardiovascular death, nonfatal myocardial infarction (MI), and coronary revascularization, and another commonly used composite “hard” endpoint. This hard endpoint includes cardiovascular death, nonfatal myocardial infarction, and absence of bloody stroke. During the 12-month median follow-up period, no significant difference was observed in the main results between the two groups (1.59% vs. 1.62%; hazard ratio, 0.41; 95% CI, 0.13–1.28). However, the absolute risk of the composite “hard” endpoint was reduced by 0.99% (0.31% vs. 1.30%; hazard ratio, 0.06; 95% CI, 0.01 to 0.53). No difference in adverse events between the two groups was observed. There is evidence that Tongxinluo capsule (TXL) can be used for reducing major adverse cardiovascular events [26]. 1,212 patients with carotid artery focal intima-media thickness (IMT) ≥ 1.2 mm were treated with TXL or placebo capsules in addition to standard therapy. The main outcome is the difference of the average annual change of IMT at 12 sites of bilateral carotid arteries over a 24-month period between the 2 groups. The secondary outcomes are differences in plaque area, vascular remodeling index (RI), serum lipids and high-sensitivity C-reactive protein levels, and a composite of the first major cardiovascular event. The results showed that the average annual changes in IMT of the TXL group and the placebo group were −0.00095 (95% CI, −0.00330 to 0.00141) mm and 0.01312 (95% CI, 0.01076 to 0.01548) mm, respectively, and the difference between the two was −0.01407 (95% CI, − 0.01740 to −0.01073) mm (*P* < 0.001). Compared with placebo, TXL treatment significantly reduced plaque area and RI, as well as the first major cardiovascular event. An RCT in Singapore randomly divided patients with cerebral infarction receiving standardized drugs and rehabilitation into NeuroAiD (Danqi Piantan capsule) group and placebo group. The results showed that NeuroAiD significantly improved the recovery of neurological function compared with placebo. However, the composite endpoint of death and vascular events and adverse drug reactions at the end of the study were not significantly different between the two groups [27]. Ginkgo biloba can improve the neurological function of patients with ischemic stroke (NIHSS score decreased by 50%, *P* < 0.05), but the study did not provide cardiovascular events or imaging data [28].

Based on the evidence above, Xinyue capsule, Suxiao Jiuxin pill, and Tongxinluo capsule can reduce the probability of dangerous events and improve symptoms and quality of life. Qing-Xin-Jie-Yu granule may be able to improve the hard endpoint of cardiovascular events, but further research and more evidence are needed. Shenzhu Guanxin recipe, Shuangshen Tongguan capsule, Xinling pill, Guanxinning tablet, Shuangshen Tongguan capsule, Danqi Piantan capsule, and Ginkgo biloba can improve the symptoms and quality of life of ASCVD patients, but there is no evidence that they can decrease the major adverse cardiovascular events.

### 3.3. Chronic Heart Failure

We included 7 studies on the treatment of chronic heart failure with traditional Chinese medicine. The number of subjects in these studies ranged from 100 to 638, and the follow-up time ranged from 5 days to 6 months. The basic characteristics of the included study are shown in Table 4. A recent study showed that Qishen Yiqi Dripping Pills (QSYQ) may be beneficial for patients with chronic ischemic heart failure (IHF) [29]. The experiment recruited 640 patients with IHF. In addition to standard treatment, patients were randomly assigned to take QSYQ or placebo for 6 months. The main outcome is a 6-minute walk in 6 months. Among the 638 IHF patients (mean age 65 years, male 72%), the 6-minute walking distance in the QSYQ group increased from 336.15 ± 100.84 meters to 374.47 ± 103.09 meters at 6 months, whereas the placebo group increased from 334.40 ± 100.27 to 340.71 ± 104.57 meters (mean changes were +38.32 and + 6.31 meters, resp.; *P* < 0.001). Compared with placebo, the secondary results of QSYQ's composite clinical events, including all-cause mortality and emergency treatment/hospitalization due to heart failure, were not significantly reduced at 6 months (13% vs. 17%; *P* = 0.45), and the brain natriuretic peptide changes of QSYQ were not significant either (median changes were −14.55 and −12.30 pg/mL, resp.; *P* = 0.21). In contrast, QSYQ's Minnesota Heart Failure Life Questionnaire score significantly improved compared with placebo (−11.78 vs. −9.17; *P* = 0.004). Adverse events of QSYQ were mild and uncommon, which were similar to the placebo group. In the context of standard treatment, Qiliqiangxin capsules further reduce the level of NT-proBNP and can improve symptoms and quality of life [30]. A total of 512 CHF patients were enrolled in the experiment, who were randomly assigned to receive placebo or Qiliqiangxin capsules on the basis of standard drug treatment. The primary endpoint is the percentage decrease or change in plasma N-terminal pro-B-type natriuretic peptide (NT-pro-BNP) levels during 12 weeks of treatment. During the 12-week follow-up, the NT-pro-BNP levels of the 2 groups were significantly lower than the baseline, but the decrease in the Qiliqiangxin capsule group was significantly higher than that of the placebo group (*P* = 0.002); in the Qiliqiangxin capsule group, 47.95% of patients had a decrease in NT-pro-BNP level by at least 30%, compared with 31.98% in the placebo group (*P* < 0.001). Qiliqiangxin capsule treatment also showed better performance than placebo in the New York Heart Association functional classification, LVEF, 6-minute walking distance, and quality of life. Shencao Tongmai Granule (STG) showed certain efficacy and safety in the treatment of CHF patients [31]. 280 CHF patients were randomly assigned to the experimental group and the control group at a ratio of 1 : 1. All patients were treated with Western medicine (WM), such as ACEI, diuretics, and digoxin elixirs. In addition, patients in the experimental group were given STG, while patients in the control group were given a placebo. The course of treatment for all patients is twelve weeks. The NYHA function classification, TCM syndrome score, and LVEF of the two groups were compared. A safety assessment was also carried out. In the end, 265 patients completed the trial (138 in the test group and 127 in the control group). The effective rate of NYHA function classification and TCM syndrome score of the experimental group was significantly higher than that of the control group (94.20% vs. 55.90% and 97.83% vs. 70.08%), and the difference was statistically significant (*P* < 0.01). There was no significant difference in LVEF between the two groups before treatment (*P* > 0.05). LVEF in both groups was increased, but the increase in LVEF after treatment in the experimental group was significantly higher than that in the control group (6.55% ±6.23% vs. 3.14% ±4.99%, *P* < 0.05). The incidence of adverse reactions in the two groups was 0.71% (1/140). The combined treatment of standard drugs and Shenmai injection (SMI) can further improve the NYHA functional classification of patients with CHF and CAD [32]. This double-blind, multicenter study randomly assigned 240 eligible patients to receive an average of SMI or placebo (100 ml/day) in addition to the standard drugs for the treatment of CHF. The primary endpoint is the functional classification of the New York Heart Association (NYHA). The secondary endpoints are 6-minute walking distance (6MWD), SF-36 scale, TCM syndrome score, left ventricular ejection fraction (LVEF), and B-type natriuretic peptide (BNP)) levels. During the first week of treatment, the NYHA functional classification of the two groups of patients gradually improved, but the SMI group improved significantly more than the placebo group (*P* = 0.001). In addition, the improvement of 6MWD, SF-36 score, and TCM syndrome score of patients receiving SMI was greater in the SMI group compared with the control group. The treatment with SMI within 1 week was well tolerated, and there were no obvious safety issues. Yangxinkang tablets improve the cardiac function, TCM syndromes, symptoms, and signs and quality of life of patients with chronic heart failure on the basis of conventional treatment and are safe and effective [33]. 228 patients with chronic heart failure of NYHA class II or III were allocated to two groups at a 1 : 1 ratio by random block method and received conventional Western medicine or conventional treatment plus Yangxinkang tablets for 4 weeks. The outcome measures were the influence of heart function before and after treatment, TCM syndrome, symptom score, physical sign score, and quality of life measured by the Minnesota Heart Failure Life Questionnaire (MLHFQ). The heart function and TCM syndromes of the two groups were improved in both groups. The total effective rate of cardiac function and TCM syndrome in the treatment group was significantly higher than that in the control group (*P* < 0.05). The total score of symptoms and signs in the treatment group decreased significantly after treatment (*P* < 0.01), which was significantly lower than that in the control group (*P* < 0.05). There were statistically significant differences in the scores of wheezing, sputum expectoration, pulmonary rales, and jugular venous distension after treatment between the two groups (*P* < 0.05 or *P* < 0.01). The three MLHFQ scores of the two groups decreased significantly after treatment (*P* < 0.01). There was no statistically significant difference between the two groups of mood subscale scores and the decline after treatment (*P* > 0.05). During the study period, no obvious adverse reactions were found in both groups. Studies have shown that Xinmailong injection (XI) can alleviate the symptoms of CHF patients, improve heart function, and improve exercise tolerance with safety [34]. The 100 CHF patients were randomly divided into XI (*n* = 50) and control group (*n* = 50). The XI group was treated with standard therapy plus XI (100 mg/2 mL, intravenous drip). The control group received standard treatment plus an equal amount of XI placebo. The course of treatment is 5 days. After the treatment, the effects of the New York Heart Association (NYHA) functional level, LVEF, BNP, and 6-minute walking distance were evaluated. The NYHA functional classification and LVEF of the XI group were better than those of the control group (*P* < 0.01). There was a significant difference in BNP between the two groups after treatment (*P* < 0.01). Compared with the control group, BNP was lower after XI treatment (*P* < 0.01). No deaths occurred during this study. There was no statistical difference in the number of adverse events between the two groups (*P* > 0.05). The results of another large clinical trial showed that Chan-Chuang Qigong can improve the exercise capacity, depression, and quality of life of patients with heart failure without harmful side effects [35].

In summary, there is evidence that Qishen Yiqi dripping pills, Qiliqiangxin capsule, Shencao Tongmai granule, Shenmai injection, Yangxinkang tablets, and Xinmailong injection can improve the symptoms and quality of life of patients with heart failure. Some studies have provided imaging evidence of improved heart function, such as Qiliqiangxin capsule and Shenmai injection. Qigong also has a certain effect on improving the quality of life. However, current research still lacks sufficient evidence to prove that traditional Chinese medicine can reduce the incidence of acute heart failure and other risk events and the rate of rehospitalization.

## 4. Discussion

Through the literature search of clinical trials of traditional Chinese medicine in recent years, we found that the scale and standardization of trials in recent years have been improved, but there are still some limitations:

1. There are various interventions in TCM, and the evidence for the efficacy of monotherapy remains insufficient. At present, there are many types of cardiovascular diseases and a variety of TCM interventions, such as traditional Chinese medicine prescriptions, Chinese patent medicines, acupuncture, moxibustion, and Qigong. In addition, we have found that in some studies, multi-intervention and multipronged approach was used to package a variety of TCM into a comprehensive treatment. The research results obtained in this way cannot clearly distinguish which treatment is effective indeed. In addition, the most common design for evaluating curative effects of TCM is the add-on design (combined use of treatments), that is, traditional Chinese medicine combined with Western medicine treatment compared with Western medicine treatment. The results can only illustrate the “tonifying” effect of traditional Chinese medicine. It is difficult to distinguish whether traditional Chinese medicine has a synergistic effect, an antagonistic effect, or an ineffective effect. Moreover, a larger sample size with prolonged periods of time is required to achieve the study goal [36, 37].
2. There are certain problems in the selection of measures for clinical outcome, which is the key to reaching clinically meaningful conclusions [38]. We found that many studies did not set clear primary and secondary outcome measures, but a list of multiple different outcome measures. The main outcome measures are usually those that have the greatest therapeutic significance and are usually set to one; otherwise, this will cause problems to interpret the results [39]. Besides, in the included studies, researchers tend to choose laboratory indicators as evaluation measures of efficacy, such as biochemical parameters in blood and radiology report, but these measures are usually surrogate outcomes. There are only a few studies report the incidence of cardiovascular events as endpoint for clinical outcome. Meanwhile, the study follow-up time for reporting endpoints is relatively short. Moreover, the efficacy evaluation criteria in many studies are based on changes in various symptoms and signs. For instance, changes in BP or symptom scores are used to determine whether interventions are effective. These assessment methods may make the conclusions inaccurate [40].
3. The safety outcome reports of the included studies are relatively simple and lack detailed description and data on potential drug interactions and other factors.

## 5. Conclusion

At present, the existing clinical trial evidence shows that some Chinese medicines may be able to effectively manage cardiovascular risk factors, such as hypertension and dyslipidemia, and treat ASCVD and chronic heart failure. However, outcome measures for the treatment of cardiovascular diseases were inconsistent, which affects the accuracy of evaluation, the secondary use of clinical trial data, and the development of clinical guideline. Therefore, it is necessary to select appropriate clinical measures which meet the research objectives and are of great significance to the disease, to further improve the value and accuracy of the research. Thus, further rigorously designed RCTs are needed to evaluate the impact of TCM on the overall mortality of patients with cardiovascular diseases or major adverse cardiovascular events.

## Figures and Tables

**Table 1:** Characteristics of included RCTs on hypertension.

Researcher	Diagnostic criteria	Sample size		Intervention		Follow-up period	Outcome
		Experiment	Control	Experiment	Control		
Dongyan Zhang	Chinese guidelines for the management of hypertension	126	125	Gastrodia-Uncaria granules	Placebo	4 weeks	Daytime and 24-hour BP decreased	
Shuhua Wang	Chinese guidelines for the management of hypertension + TCM syndrome differentiation	160	80	Tiankuijiangya tablet + standard treatment	Placebo + standard treatment	8 weeks	Reduction of BP	
Chunxiao Wu	Chinese guidelines for the management of hypertension	100	50	Bushen Qinggan Formula + standard treatment	Placebo + standard treatment	8 weeks	BPD and BPV decreased	
Jinpeng Ma	Chinese guidelines for the management of hypertension	54	64	Qiqilian Capsule + standard treatment	Placebo + standard treatment	4 weeks	Reduction of BP	
Xudong Liu	Chinese guidelines for the management of hypertension + TCM syndrome differentiation	52	51	Jingui Shenqi pill + standard treatment	Standard treatment	6 weeks	Reduction of BP	
Xi Chen	Chinese guidelines for the management of hypertension + TCM syndrome differentiation	99	100	Jiangyabao tablet + standard treatment	Placebo + standard treatment	24 weeks	Reduction of BP	
Hui Zheng	Chinese guidelines for the management of hypertension + JNC‐7	107(2 experiment groups)	107(2 control groups)	Affected acupuncture point, nonaffected acupuncture point	Sham acupuncture, waiting‐list control	12 weeks	Minor reduction of BP	

**Table 2:** Characteristics of included RCTs on dyslipidemia.

Researcher	Diagnostic criteria	Sample size		Intervention		Follow-up period	Outcome
		Experiment	Control	Experiment	Control		
Ying Xie	Chinese guidelines on prevention and treatment of dyslipidemia in adults	69	69	Jiangzhi Tongluo soft capsule + standard treatment	Placebo + standard treatment	8 weeks	Higher decreased value of TG
Timothy Kwok	LDL ≥ 3.5 mmol/L and <4.9 mmol/L	85	80	D&G capsule	Placebo	12 months	Higher decrease in LDL and TC,
Wenhao Jia	TG > 2.3 mmol/L but <6.5 mmol/L),LDL-C < 4.9 mmol/L, total cholesterol (TC) < 7.2 mmol/L	285(2 experiment groups)	73	XZT, XZK	Placebo	12 weeks	Increases in HDL, decreased TG level
Long Liu	TC ≥ 5.2 mmol/L or TG ≥ 1.65 mmol/L	60	60	*Ganoderma lucidum* and sea cucumber extract	Placebo	45 days	Decreased TC and TG level
Bo Wu	Acupuncture treatment of obesity + TCM syndrome differentiation	52	52	Acupuncture + tapping	Acupuncture	3 months	Improved blood lipid level
Syed Kazem Farahmand	Metabolic syndrome, TG ≥ 1.7 mmol/L	63	63	Dietary regimen + wet cupping treatment	Dietary regimen	6 weeks	No significant difference in blood lipids

**Table 3:** Characteristics of included RCTs on ASCVD.

Researcher	Diagnostic criteria	Sample size		Intervention		Follow-up period	Outcome
		Experiment	Control	Experiment	Control		
Jianwei Gao	Guideline for diagnosis and treatment of patients with chronic stable angina, ACC/AHA/ACP–ASIM guidelines for the management of chronic stable Angina + positive result of coronary angiography or CTA or MPI+	115	117	Xinling Wan pill	Placebo	4 weeks	Reduced the amount of nitroglycerin, relieved symptoms
Junbo Ge	After AMI or PCI or CABG, epicardial coronary stenosis of ≥50% in at least one major branch	1335	1327	Shexiang Baoxin pill + optimal medical therapy	Placebo + optimal medical therapy	24 months	Reduced the occurrence of MACEs, reduced angina frequency
Ming Guo	After PCI	530	524	Xinyue capsule + standard treatment	Placebo + standard treatment	1 year	Reduced occurrence of primary endpoint event
Zhijie Shen	Guidelines for the diagnosis and treatment of unstable angina pectoris and non-ST-segment Elevation myocardial infarction + guidelines for the diagnosis and treatment of acute ST-elevation myocardial infarction	92	95	Suxiao Jiuxin pill + standard treatment	Placebo + standard treatment	12 months	Reduced the occurrence of MACEs, improved LVEF and SAQ
Wang Yonggang	After PCI + TCM syndrome differentiation	67	62	Shuangshen Tongguan capsule + standard treatment	Placebo + standard treatment	6 months	Improved LVEF and SAQ, increased *k*-value of the microcirculation perfusion
Danping Xu	ACC/AHA guidelines for the management of patients with chronic stable angina + TCM syndrome differentiation	59	55	Shenzhu Guanxin recipe + standard treatment	Placebo + standard treatment	90 days	Improved SAQ and daily exercise tolerance, reduced the amount of nitroglycerin
Jingen Li	Patients with SCAD	750	750	Qing-Xin-Jie-Yu Granule + standard treatment	Placebo + standard treatment	12 months	Reduced risk of the composite “hard” endpoint
Mei Zhang	Patients with noncalcified plaque	607	605	Tongxinluo capsule + standard treatment	Placebo + standard treatment	24 months	Reduced IMT and major cardiovascular events
Narayanaswamy Venketasubramanian	Ischemic stroke of intermediate severity (NIHSS) in the preceding 72h, neuroimaging findings compatible with cerebral infarction and mRS ≤1.	550	549	NeuroAiD (Danqi Piantan capsule) + standard treatment	Placebo + standard treatment	24 months	No obvious difference in rates of death and occurrence of vascular and other medical events
Darioush Savadi Oskouei	AIS involving anterior cerebral circulation	52	50	Ginkgo biloba	Placebo	4 months	Reduced NIHSS score

**Table 4:** Characteristics of included RCTs on chronic heart failure.

Researcher	Diagnostic criteria	Sample size		Intervention		Follow-up period	Outcome
		Experiment	Control	Experiment	Control		
Jingyuan Mao	Ischemic heart disease with LVEF ≤45%, NYHA II–IV	319	319	Qishen Yiqi dripping pills + standard treatment	Placebo + standard treatment	6 months	Increased 6-minute walking distance, improved the quality of life
Xinli Li	Chinese guidelines for the diagnosis and management of CHF, LVEF ≤ 40% and a serum NT-proBNP level 450 pg/ml	256	256	Qili Qiangxin capsules + standard treatment	Placebo + standard treatment	12 weeks	Reduced NT-proBNP level, improved NYHA functional classification, LVEF and 6-min walking distance
Chen Wang	The Framingham HF diagnostic criteria + TCM syndrome differentiation	138	127	Shencao Tongmai granule + standard treatment	Placebo + standard treatment	12 weeks	Improved NYHA functional classification and LVEF
Shaoxiang Xian	CHF combined with CAD, NYHA functional classification II–IV + TCM syndrome differentiation	120	120	Shenmai injection + standard treatment	Placebo + standard treatment	7 days	Improved NYHA functional classification, and 6-minute walking distance and TCM syndrome score
Shao-Xiang Xian	Clinical cardiology, 3200 medical Disease diagnostic Criteria + TCM syndrome differentiation	116	112	Yangxinkang tablets + standard treatment	Placebo + standard treatment	4 weeks	Improved the quality of life and symptoms
Jingui Xue	ACCF/AHA guidelines for the diagnosis and management of heart failure in adults	50	50	Xinmailong injection + standard treatment	Placebo + standard treatment	5 days	Reduced BNP, improved NYHA functional classes and LVEF
Ju-Hsin Cheng	AHA guidelines for the diagnosis and management of heart failure in adults, NYHA functional classification II	50	50	Chan-Chuang Qigong	Blank control	12 weeks	Improved the quality of life and 6-minute walking distance

## Supplementary Materials

Supplementary Materials Table 1: characteristics of included RCTs on hypertension. Table 2: characteristics of included RCTs on dyslipidemia. Table 3: characteristics of included RCTs on ASCVD. Table 4: characteristics of included RCTs on chronic heart failure.
