# Supplementary material for: Current Status and Evaluation of Randomized Clinical Trials of Traditional Chinese Medicine in the Treatment of Cardiovascular Diseases
Source: Evid Based Complement Alternat Med. 2022 Jan 12;2022:6181862. doi: 10.1155/2022/6181862 (PMC8769817; doi:10.1155/2022/6181862)
Supplement: Supplementary Materials — Table 1: characteristics of included RCTs on hypertension. Table 2: characteristics of included RCTs on dyslipidemia. Table 3: characteristics of included RCTs on ASCVD. Table 4: characteristics of included RCTs on chronic heart failure. [file 6181862.f1.docx]

**Table 1, Characteristics of included RCTs on hypertension**

| Researcher | Diagnostic criteria | Sample size | | Intervention | | | Follow-up period | | Outcome |
| --- | --- | --- | --- | --- | --- | --- | --- | --- | --- |
|  |  | Experiment | control | Experiment | | control |  |  |  |
| Dongyan Zhang | Chinese Guidelines for the Management of Hypertension | 126 | 125 | Gastrodia-uncaria granules | Placebo | | 4 weeks | Daytime and 24-hour BP decreased | |
| Shuhua Wang | Chinese Guidelines for the Management of Hypertension +TCM syndrome differentiation | 160 | 80 | Tiankuijiangya tablet+standard treatment | Placebo+standard treatment | | 8 weeks | Reduction of BP | |
| Chunxiao Wu | Chinese Guidelines for the Management of Hypertension | 100 | 50 | Bushen Qinggan Formula+standard treatmen | Placebo+standard treatment | | 8 weeks | BPD and BPV decreased | |
| Jinpeng Ma | Chinese Guidelines for the Management of Hypertension | 54 | 64 | Qiqilian Capsule+ standard treatment | Placebo+ standard treatmen | | 4 weeks | Reduction of BP | |
| Xudong Liu | Chinese Guidelines for the Management of Hypertension +TCM syndrome differentiation | 52 | 51 | Jingui Shenqi pill + standard treatment | standard treatment | | 6 weeks | Reduction of BP | |
| Xi Chen | Chinese Guidelines for the Management of Hypertension +TCM syndrome differentiation | 99 | 100 | Jiangyabao tablet + standard treatment | Placebo+ standard treatmen | | 24 weeks | Reduction of BP | |
| Hui Zheng | Chinese Guidelines for the Management of Hypertension + JNC‐7 | 107（2 experiment groups） | 107(2 control groups) | Affected acupuncture point, non-affected acupuncture point | Sham acupuncture, waiting‐list control | | 12weeks | Minor reduction of BP | |

| Researcher | Diagnostic criteria | Sample size | | | Intervention | | | Follow-up period | | Outcome |
| --- | --- | --- | --- | --- | --- | --- | --- | --- | --- | --- |
|  |  | Experiment | | control | Experiment | | control |  |  |  |
| Ying xie | Chinese guidelines on Prevention and Treatment of Dyslipidemia in Adults | 69 | 69 | | jiangzhi tongluo soft capsule +standard treatment | Placebo+standard treatment | | 8 weeks | Higher decreased value of TG | |
| Timothy Kwok | LDL≥3.5mmol/L and <4.9 mmol/L | 85 | 80 | | D&G capsule | Placebo | | 12 months | Higher decrease in LDL and TC, | |
| Wenhao Jia | TG> 2.3 mmol/L but <6.5 mmol/L),LDL-C < 4.9 mmol/L, total cholesterol (TC) < 7.2 mmol/L | 285(2 experiment groups) | 73 | | XZT,XZK | Placebo | | 12 weeks | Increases in HDL, decreased TG level | |
| Long Liu | TC≥5.2 mmol /L or TG≥1.65 mmol /L | 60 | 60 | | Ganoderma lucidum and sea cucumber extract | Placebo | | 45 days | Decreased TC and TG level | |
| Bo Wu | Acupuncture treatment of Obesity +TCM syndrome differentiation | 52 | 52 | | Acupuncture+tapping | Acupuncture | | 3 months | Improved blood lipid level | |
| Syed Kazem Farahmand | metabolic syndrome，TG≥1.7mmol/L | 63 | 63 | | Dietary regimen + wet-cupping treatment | dietary regimen | | 6 weeks | No significant difference in blood lipids | |

**Table 2, Characteristics of included RCTs on dyslipidemia**

**Table 3, Characteristics of included RCTs on ASCVD**

| Researcher | Diagnostic criteria | Sample size | | | Intervention | | | Follow-up period | | Outcome |
| --- | --- | --- | --- | --- | --- | --- | --- | --- | --- | --- |
|  |  | Experiment | | control | Experiment | | control |  |  |  |
| Jianwei Gao | Guideline for diagnosis and treatment of patients with chronic stable angina, ACC/AHA/ACP–ASIM Guidelines for the Management of Chronic Stable Angina+Positive result of coronary angiography or CTA orMPI+ | 115 | 117 | | Xinling Wan Pill | Placebo | | 4 weeks | Reduced the amount of nitroglycerin, relieved symptoms | |
| Junbo Ge | After AMI or PCI or CABG, epicardial coronary stenosis of ≥ 50% in at least one major branch | 1335 | 1327 | | Shexiang Baoxin pill+ optimal medical therapy | Placebo+ optimal medical therapy | | 24months | Reduced the occurrence of MACEs, reduced angina frequency | |
| Ming Guo | After PCI | 530 | 524 | | Xinyue Capsule+standard treatment | Placebo+standard treatment | | 1 year | Reduced occurrence of primary endpoint event | |
| Zhijie Shen | Guidelinesfor the Diagnosis and Treatment of Unstable Angina Pectoris and Non-ST-Segment Elevation Myocardial Infartion+Guidelines for the diagnosis and treatment of acute ST-elevation myocardial infarction | 92 | 95 | | Suxiao Jiuxin Pill+standard treatment | Placebo+standard treatment | | 12 months | Reduced the occurrence of MACEs, improved LVEF and SAQ | |
| Wang yonggang | After PCI+ TCM syndrome differentiation | 67 | 62 | | Shuangshen Tongguan Capsule+standard treatment | Placebo+standard treatment | | 6 months | Improved LVEF and SAQ, increased k value of the microcirculation perfusion | |
| Danping Xu | ACC/AHA guidelines for the management of patients with chronic stable angina + TCM syndrome differentiation | 59 | 55 | | Shenzhu Guanxin Recipe+ standard treatment | Placebo+standard treatment | | 90 days | Improved SAQ and Daily exercise tolerance, reduced the amount of nitroglycerin | |
| Jingen Li | Patients with SCAD | 750 | 750 | | Qing-Xin-Jie-Yu Granule+ standard treatment | Placebo+ standard treatment | | 12 months | Reduced risk of the composite ‘hard’ endpoint | |
| Mei Zhang | Patients with non-calcified plaque | 607 | 605 | | Tongxinluo capsule+ standard treatment | Placebo+ standard treatment | | 24 months | Reduced IMT and major cardiovascular events | |
| Narayanaswamy Venketasubramanian | Ischemic stroke of intermediate severity（NIHSS）in the preceding 72h, neuroimaging findings compatible with cerebral infarction  and mRS ≤ 1. | 550 | 549 | | NeuroAiD (DanqiPiantan Capsule)+ standard treatment | Placebo+ standard treatment | | 24 months | No obvious difference in rates of death and occurrence  of vascular and other medical events | |
| Darioush Savadi Oskouei | AIS involving anterior cerebral circulation | 52 | 50 | | Ginkgo biloba | Placebo | | 4 months | Reduced NIHSS score | |

**Table 4, Characteristics of included RCTs on chronic heart failure**

| Researcher | Diagnostic criteria | Sample size | | | Intervention | | | Follow-up period | | Outcome |
| --- | --- | --- | --- | --- | --- | --- | --- | --- | --- | --- |
|  |  | Experiment | | control | Experiment | | control |  |  |  |
| Jingyuan Mao | Ischaemic heart disease with LVEF ≤ 45%, NYHA II–IV | 319 | 319 | | Qishen Yiqi dripping pills+ standard treatment | Placebo+standard treatment | | 6 months | Increased 6 min walking distance, improved the quality of life | |
| Xinli Li | Chinese guidelines for the diagnosis and management of CHF, LVEF≤ 40% and  a serum NT-proBNP level 450 pg/ml | 256 | 256 | | Qili Qiangxin Capsules+ standard treatment | Placebo+standard treatment | | 12 weeks | Reduced NT-proBNP level, improved NYHA functional classification, LVEF and 6-min walking distance. | |
| Chen Wang | The Framingham HF Diagnostic Criteria +TCM syndrome differentiation | 138 | 127 | | Shencao Tongmai Granule+ standard treatment | Placebo+standard treatment | | 12 weeks | Improved NYHA functional classification and LVEF | |
| Shaoxiang Xian | CHF combined with CAD, NYHA functional classification II–IV+ TCM syndrome differentiation | 120 | 120 | | Shenmai injection+ standard treatment | Placebo+standard treatment | | 7 days | Improved NYHA functional classification, and 6-min walking distance and TCM syndrome score | |
| Shao-xiang Xian | Clinical Cardiology, 3200 Medical  Disease Diagnostic Criteria+ TCM syndrome differentiation | 116 | 112 | | Yangxinkang Tablets+ standard treatment | Placebo+standard treatment | | 4 weeks | Improved the quality of life and symptoms. | |
|  |  |  |  | |  |  | |  |  | |
|  |  |  |  | |  |  | |  |  | |
| Jingui Xue | ACCF/AHA Guidelines for the Diagnosis and Management of Heart Failure in Adults | 50 | 50 | | Xinmailong Injection+ standard treatment | Placebo+standard treatment | | 5 days | Reduced BNP, improved NYHA functional classes and LVEF | |
| Ju-Hsin Cheng | AHA Guidelines for the Diagnosis and Management of Heart Failure in Adults, NYHA functional classification II | 50 | 50 | | Chan-Chuang Qigong | Blank control | | 12 weeks | Improved the quality of life and 6-min walking distance | |
|  |  |  |  | |  |  | |  |  | |
